# Evidence for Gene-Specific Rather Than Transcription Rate–Dependent Histone H3 Exchange in Yeast Coding Regions

**DOI:** 10.1371/journal.pcbi.1000282

**Published:** 2009-02-06

**Authors:** Irit Gat-Viks, Martin Vingron

**Affiliations:** Computational Molecular Biology Department, Max Planck Institute for Molecular Genetics, Berlin, Germany; University of Washington, United States of America

## Abstract

In eukaryotic organisms, histones are dynamically exchanged independently of DNA replication. Recent reports show that different coding regions differ in their amount of replication-independent histone H3 exchange. The current paradigm is that this histone exchange variability among coding regions is a consequence of transcription rate. Here we put forward the idea that this variability might be also modulated in a gene-specific manner independently of transcription rate. To that end, we study transcription rate–independent replication-independent coding region histone H3 exchange. We term such events *relative exchange*. Our genome-wide analysis shows conclusively that in yeast, relative exchange is a novel consistent feature of coding regions. Outside of replication, each coding region has a characteristic pattern of histone H3 exchange that is either higher or lower than what was expected by its RNAPII transcription rate alone. Histone H3 exchange in coding regions might be a way to add or remove certain histone modifications that are important for transcription elongation. Therefore, our results that gene-specific coding region histone H3 exchange is decoupled from transcription rate might hint at a new epigenetic mechanism of transcription regulation.

## Author Summary

During nucleosome disassembly and reassembly, evicted histones are exchanged with newly synthesized histones. Histone exchange occurs in several DNA metabolism processes, including replication, transcription, and repair. Recent reports from several labs show that replication-independent histone H3 exchange in yeast coding regions is tightly correlated with transcription rate. We have computationally shown that histone exchange variability among genes is not only a consequence of transcription rate. Instead, each coding region has a characteristic amount of replication-independent histone exchange, even when excluding the confounding effect of transcription rate. We show that this transcription rate–independent exchange, referred to as *relative exchange*, is a reproducible and consistent feature of the entire coding region and cannot be explained by regional effects. Next, we characterize the relations between relative exchange and a variety of histone H3 modifications, as well as the histone chaperone Asf1. Taken together, our analysis shows that gene-specific replication-independent histone H3 exchange in coding regions is mediated independently of transcription rate, thus constituting a new mechanism in epigenetic transcription regulation.

## Introduction

The nucleosome is the basic repeating unit of the chromatin and comprises 147 bp of DNA wrapped around an octamer of histone proteins (two copies of H2A, H2B, H3 and H4). Nucleosome disassembly and reassembly is tightly coupled with replication, transcription, DNA repair and heterochromatin silencing (e.g., [1],[2]). Under normal circumstances, histones are associated with specific histone chaperones that assist their assembly and disassembly [3]. During disassembly and reassembly of nucleosomes, the original histones might be exchanged (replaced) by newly synthesized histones [4]–[8]. Outside of replication, histone H3 exchange occurs predominantly at promoters, whereas H3 exchange in coding regions is significantly lower [7],[8].

Coding region replication-independent H3 exchange varies from gene to gene. Recent studies have shown that this variation among coding region is linked to differences in transcription rate [6]–[9]. For example, genome-wide studies demonstrate a strong association between transcription rate and replication-independent histone H3 exchange in yeast coding regions 7,8. This association is expected: During transcription elongation, nucleosomes are disassembled in front of the elongating RNA polymerase II (RNAPII) complex to enable its passage, and reassemble almost immediately behind it (reviewed by [10]–[12]). Behind RNAPII, the original H3/H4 histones might be either exchanged (replaced) or retained (not replaced; [13]). Therefore, the amount of coding region H3 exchange is expected to reflect the number of transcripts produced by RNAPII.

Although it is widely accepted that coding region replication-independent H3 exchange differences are a consequence of transcription rate, it is still unknown whether this variability is also controlled independently of transcription rate. In other words, it is not clear whether different coding regions can have substantially different replication-independent H3 exchange even if they have the same transcription rate. This leads us to investigate genome-wide coding region H3 exchange independently of both replication and transcription rate. We address two key questions: First, is there evidence that transcription rate–independent and replication-independent H3 exchange in coding regions is a consistent feature of genes? Second, is there evidence for an active regulation of this feature?

In this study, we analyzed published data sets of replication-independent histone H3 exchange in yeast [7],[8]. The measured amount of replication-independent histone H3 exchange is simply called *total exchange*, whereas the calculated transcription rate–independent total exchange is referred to as *relative exchange*. Positive (negative) relative exchange implies that the total exchange is higher (lower) than what is expected based on transcription rate alone. Importantly, although relative exchange is independent of transcription rate, it is still likely to be influenced by the transcription process. We found that relative exchange varies from gene to gene and is a reproducible feature of genes. Elevated or reduced relative exchange occurs along the entire coding region and not only in a specific part of it. Moreover, relative exchange is a gene-specific property rather than a regional effect. Finally, we revealed that H3K79 trimethylation is depleted in coding regions with hyper relative exchange and enriched in coding regions with hypo relative exchange. Taken together, our data provides evidence that coding regions have a characteristic relative exchange, a new feature of genes. Genes might have either hyper or hypo relative exchange, irrespective of their total exchange or transcription rate. Histone exchange in coding regions might be a way to add or remove certain histone modifications that are important for transcription elongation. Therefore, decoupling replication-independent histone exchange from transcription rate is a process with potential for epigenetic gene regulation.

Asf1 is a histone H3/H4 chaperone that has been implicated in histone H3/H4 exchange during elongation [4],[14]. Recently it was shown that outside of replication, Asf1-mediated H3 exchange globally correlates with the amount of total exchange and with transcription rate [8]. In addition to Asf1's role in histone exchange, Asf1 is also known as a global regulator of gene expression [15]. Interestingly, many genes are down-regulated by Asf1, whereas other genes are up-regulated by its influence. This dual function of Asf1 as specific negative and positive regulator of gene expression is well documented [2],[14],[15], but is still largely unexplained. Here we show a global association between Asf1-mediated gene expression and relative exchange (but no direct association with total exchange). Genes with hyper relative exchange tend to be down regulated by Asf1, whereas genes with hypo relative exchange are up-regulated by Asf1. Therefore, the relative exchange property provides insights into the longstanding question as to the selective positive and negative transcriptional influence of Asf1.

## Results

### Replication-Independent and Transcription Rate–Independent Histone H3 Exchange in Coding Regions

The present work is focused on the understanding of replication-independent histone H3 exchange in coding regions. For our study, we used published genome-wide measurements of histone H3 exchange and RNAPII densities [7],[8]. The data was taken from G1-arrested cells, hence eliminating the contribution of histone exchange during replication. In the following, *transcription rate* is defined as RNAPII density averaged over the coding region. *Total exchange* is the measured replication-independent histone H3 exchange averaged over the coding region.

**Figure 1:**
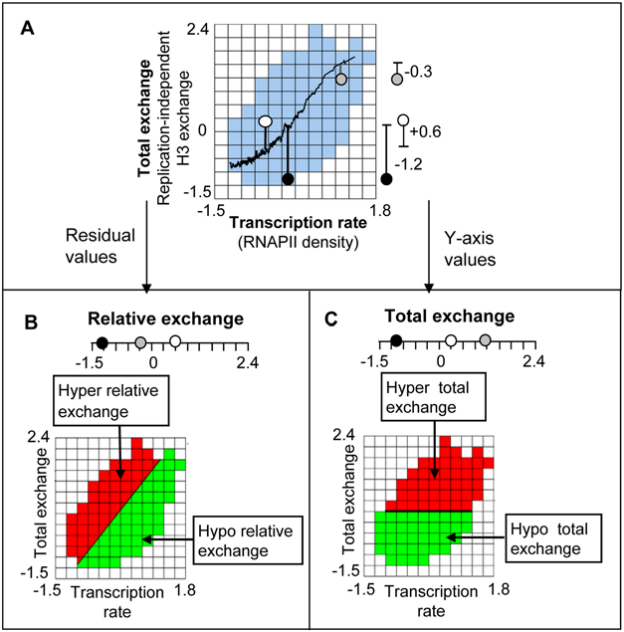
An illustration of relative exchange vs. total exchange. (A) A heat map illustrating the functional relationship between transcription rate (*x*-axis) and total exchange (*y*-axis; [8]). Transcription rate is the average RNAPII density over the coding region, whereas total exchange is the replication-independent histone H3 exchange averaged over the coding region. Each cell represents a 2D bin including all genes with total exchange and transcription rate in a defined range. The color of the bin represents the number of genes contained within it. Empty and near-empty 2D bins (below five genes) are colored white, whereas bins with more then five genes are colored blue. The heat map clearly shows that total exchange correlates with transcription rate (as previously observed by [7],[8], see Figure S1 in Text S1). To analyze total exchange without the confounding effect of transcription rate, we introduced relative exchange. A coding region's *relative exchange* is the difference between its total exchange and a running average of total exchange (the running average is marked as a black curve). For example, the heat map illustrates three coding regions marked in black, white, and gray circles, whose total exchange values are −1.1, +0.2, and +1.2 (C), but their amount of relative exchange is −1.2, +0.6, and −0.3, respectively (B). Bottom panels: Red/green represents bins with positive/negative value of total exchange (C) or relative exchange (B).

Recent reports show that outside of replication, there is a clear correlation between coding region replication-independent histone H3 exchange and RNAPII density (Figure 1A, and Figure S1 in Text S1; [6]–[8]). Beyond this global relationship, there is a wide distribution around the diagonal. Hence, even at the same transcription rate, different genes differ in their amount of replication-independent histone H3 exchange. To determine whether this variation has a biological basis, we extracted and interpreted this information in a systematic manner as follows: *Relative exchange* is the distance of total exchange to a running average of the total exchange along the transcription rate axis (Figure 1B). In case that we analyze relative exchange of each single tiling-array probe (denoted *probe's relative exchange*), we used measurements of total exchange and RNAPII density in a single probe without averaging over the entire coding region (see Methods). The calculated relative exchange values eliminate the contribution of transcription rate from the total exchange in coding regions. Relative exchange is substantially different and is not monotonic with the amount of total exchange (see an illustrative example in Figure 1). Total exchange is replication-independent, whereas relative exchange is replication-independent and transcription rate–independent.

**Figure 2:**
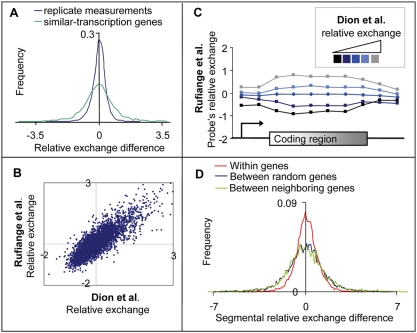
Relative exchange is a gene-specific property. (A) Distribution of relative exchange difference between genes with similar transcription rate (*similar-transcription genes*, green; [8]) and between replicate measurements (blue; [8]). The plot demonstrates that relative exchange differences are only partially explained by experimental noise. (B) A scatter plot showing the relationship between relative exchange calculated based on data from Dion et al. (*x*-axis, [7]) and Rufiange et al. (*y*-axis, [8]). The strong correlation indicates that total exchange is still informative even when eliminating the contribution of transcription rate. (C) The graphs represent composite profiles of probe's relative exchange from Rufiange et al., which were divided into five groups according to their relative exchange based on Dion et al. (shown in shades of blue, [7]). The length of the coding region was divided into six segment bins of equal length and the probes were assigned according to their nearest corresponding relative position. Outside of the coding region, aligned probes were assigned to 50 bp segment bins. Presented are the probe's relative exchange, averaged over all probes at the same group and the same segment bin. The plot demonstrates that relative exchange is reproducible in each small segment of the coding region. (D) The distribution of relative exchange differences between segments of the same coding region (red, denoted *within genes*; see Methods), between neighboring and between random genes (green and blue, respectively; presented are distances between the first segments). The variance within genes is smaller than the variance between neighboring or random genes, indicating that relative exchange is consistent along the coding region. Moreover, random and neighboring genes have similar distribution, indicating that relative exchange is a gene-specific property rather than a regional effect.

The observed relative exchange variation among genes might be a consequence of biological or experimental noise. To exclude the latter, we grouped genes into pairs with minimal difference in transcription rate. Such a gene pair is termed *similar-transcription genes*. The relative exchange difference between two similar-transcription genes was calculated by subtracting the relative exchange of the gene whose transcription rate is lower from the paired (higher transcription rate) gene. For comparison, we have computed the difference between relative exchange replicates (calculated based on replicates from [8]). Figure 2A demonstrates that the distribution of relative exchange differences between similar-transcription genes is broader than the distribution of differences between replicates, indicating that experimental noise can only partially account for relative exchange variation [F-test P<10^−200^ (F-test for significance of difference between variances)]. This observation is particularly significant because of the bias toward both negative and positive differences between similar-transcription genes. Whereas the bias toward positive values can be attributed to the global relationships with transcription rate, this effect cannot explain the bias toward negative values.

Next, we have investigated the reproducibility of relative exchange in different laboratories. Total exchange (together with transcription rate) was measured in two different laboratories [7],[8]. Therefore, we asked whether relative exchange calculated based on measurement from Rufiange et al. correlates with relative exchange based on Dion et al. Our rationale is that if the relative values are only noise, there will be a poor correlation between relative exchange values taken from two different laboratories. On the other hand, if relative exchange is informative, measurements from the two laboratories should show good correlation. Remarkably, we found that relative exchange measured by Rufiange et al. exhibit good correlation with relative exchange measured by Dion et al. (Spearman correlation = 0.84, P-value<10^−200^, Figure 2B). The correlation among relative exchange from the two laboratories is almost as good as the correlation among the measured total exchange replicates (Spearman correlation = 0.85). We validated that this reproducibility is not a byproduct of (i) a bias in total exchange or RNAPII density measurements, (ii) using average RNAPII density as an approximation of transcription rate, (iii) GC-content and sequence properties, (iv) averaging genes of different lengths (see Text S1 for details). Taken together, Figure 2A and 2B suggest that total exchange is informative even after eliminating the contribution of transcription rate (for a quantitative evaluation, see Text S2).

### Relative Exchange Is a Gene-Specific Property of Coding Regions

To examine whether relative exchange is a general property of genes, we wished to analyze the reproducibility of relative exchange along the entire coding region. To that end, we divided the genes into five subsets according to their relative exchange based on Dion et al. For each subset, we plotted a profile of probe's relative exchange throughout the coding region based on Rufiange et al. (Figure 2C). Note that the probe's relative exchange values were calculated based on measurements in single probes and were not averaged over the coding region (see above and Methods). We observe that the reproducibility of probe's relative exchange is found in each small segment of the coding region.

**Figure 3:**
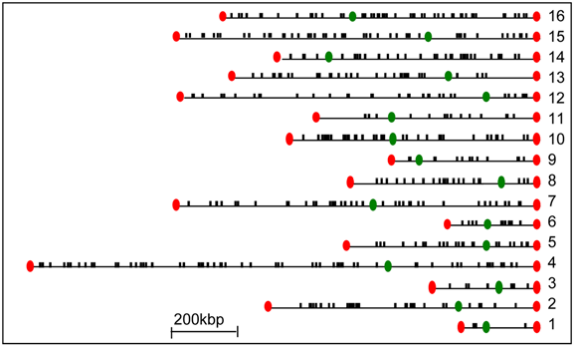
Chromosomal distribution of the 500 coding regions with the highest relative exchange (relative exchange>0.7).

We next considered the consistency of relative exchange along the coding region. To that end, we have split each coding region into six segments of equal length. The relative exchange of a segment (denoted *segmental relative exchange*) was calculated using only probes located within this segment (see Methods). Figure 2D shows that relative exchange differences between segments of the same coding region (denoted *within genes*) tend to be smaller than relative exchange differences between segments from neighboring genes (denoted *between genes*). In agreement, relative exchange variation between genes is significantly larger than the variation within genes [P<10^−200^ (F-test for significance of difference between variances)], indicating the consistency of relative exchange along the coding region. Notably, the distribution of relative exchange differences between neighboring genes is similar to the distribution of relative exchange differences between random genes (Figure 2D). The same comparison on total exchange (rather than relative exchange) gives similar results (Figure S2 in Text S2). Taken together, it appears that each gene has a characteristic relative exchange along the coding region, and relative exchange is a gene-specific property rather than a regional effect. Some coding regions have *hyper relative exchange* (or *hypo relative exchange*), based on their total exchange that is relatively higher (or lower) from what can be expected from their transcription rate alone. A genome-wide mapping shows that hyper relative exchange coding regions are scattered throughout the genome (Figure 3).

### Relation between Chromatin Modifications and Relative Exchange

**Figure 4:**
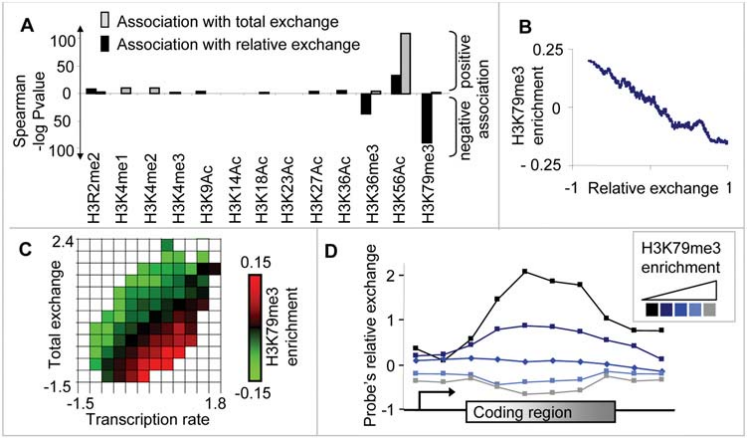
H3K79 trimethylation in coding regions is tightly linked to relative exchange. (A) A histogram showing the correlation between each histone H3 modification (*x*-axis; Text S3) and two histone exchange measures: relative exchange (black) or total exchange (gray) in coding regions. *y*-Axis: −log P-value of Spearman correlation (see Methods). Positive/negative correlations appear above/below the *x*-axis. Among all histone marks, H3K79me3 has the strongest association with relative exchange. (B) Plot of the relationship among H3K79me3 enrichment against relative exchange in coding regions. The plot was generated using a moving average (window = 100, step = 1). H3K79me3 enrichment is the log2 ratio of H3K79me3 ChIP vs. anti-H3 ChIP [17]. (C) A heat map illustrating the functional relationship between transcription rate (*x*-axis, [8]), total exchange in coding regions (*y*-axis, [8]), and H3K79me3 enrichment in coding regions (color-coded). The heat map is depicted as in Figure 1A, except that the color-coding is different (see Methods). High/low H3k79me3 enrichment is indicated in red/green. The heat map demonstrates that H3K79me3 is linked to relative exchange but not to total exchange. (D) Composite profiles of probe's relative exchange along the coding region. Coding regions were divided into five groups according to their average H3K79me3 enrichment (shown in shades of blue). The plot is depicted as in Figure 2C, except that the partition into groups is different. We observe that the link between H3K79me3 enrichment and relative exchange holds along the entire coding region.

To systematically characterize relative exchange, we compared it with published genome-wide profiles of histone H3 modifications (data sources are [8], [16]–[19]; see Text S3 for details). The analysis was limited to coding regions. Figure 4A demonstrates the correlation between each modification and relative exchange vis-à-vis the correlation with total exchange. For each modification, we calculated its average enrichment in each coding region, and compared it with the (average) relative or total exchange of the coding regions. To avoid complications arising from averaging on coding regions with different lengths, we used Spearman correlation calculated independently of transcript length. Further, the total exchange was factored out from the correlation with relative exchange and vise versa (see Methods). In agreement with previous observations [8], total exchange is mainly associated with H3K56 acetylation (H3K56Ac; see the table Figure S3 in Text S3).

Unexpectedly, we found that relative exchange is tightly related to H3K79 trimethylation (H3K79me3 [17]). H3K79me3 anti-correlates with relative exchange (Spearman correlation = −0.42, P-value = 10^−88^, Figure 4A and 4B) but not with total exchange (Spearman correlation = 0.01, P-value = 10^−1^), indicating that H3K79me3 is specifically associated with relative exchange. In agreement, Figure 4C clearly demonstrates that H3K79me3 is linked to relative exchange rather than to total exchange (compare with the bottom panel of Figure 1B). The association holds throughout the entire coding region (Figure 4D). We obtained the same results when the association is computed independently of GC content (data not shown). We conclude that the pattern of H3K79me3 is related to relative exchange.

Previous studies show that in coding regions, there is a correlation between H3K79me3 and transcription rate (e.g., [17]). We asked whether this correlation holds even when eliminating the effect of histone exchange. Interestingly, the general correlation between H3K79me3 and transcription rate (Spearman correlation = 0.19) becomes much higher when eliminating the effect of histone exchange (total exchange-independent Spearman correlation = 0.46). This demonstrates that any genome-wide analysis of H3K79me3 must take into consideration the effect of histone H3 exchange.

Histone H3K36 can be a target of acetylation, mono-, di- and trimethylation (Ac, me, me2, me3, respectively). Recent report shows that H3K36Ac pattern is inversely related to H3K36me2 and H3K36me3 patterns in coding regions, suggesting that H3K36 is an ‘acetyl/methyl switch’ [19]. Here we found that relative exchange is significantly associated with H3K36me3 (Spearman correlation = −0.28, P-value = 10^−35^, Figure S4 in Text S4), but the association with H3K36Ac is significantly lower (Spearman correlation P-value = 10^−3^). Therefore, our analysis indicates that although both H3K36me3 and H3K36Ac are inversely related, they are associated differentially with relative exchange.

### Asf1-Mediated Exchange Is Linked to Total Exchange

**Figure 5:**
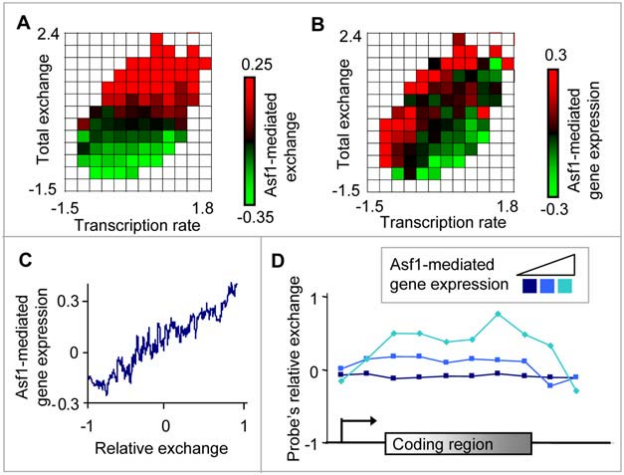
Relative exchange is linked to Asf1's transcriptional influence. (A,B) A heat map illustrating the functional relationship between transcription rate (*x*-axis, [8]), total exchange in coding regions (*y*-axis, [8]), and a color-coded Asf1 influence. The heat maps are depicted as in Figure 1A (see Methods), except that the color coding is different. Plot A is color-coded by Asf1-mediated exchange [8]. Higher/lower dependency of total exchange on Asf1 is in red/green. Plot B is color-coded by Asf1-mediated gene expression [15]. Down/up-regulation of Asf1 on its transcriptional targets is indicated in red/green. Asf1-mediated exchange is linked to total exchange (A) (previously reported by [8], but surprisingly Asf1-mediated gene expression is linked to relative exchange (B). (C) Plot of the relationship among Asf1-mediated gene expression against relative exchange in coding regions. The graph shows a moving average (window = 100, step = 1) of the Asf1-mediated gene expression over relative exchange. (D) Composite profiles of probe's relative exchange along the coding region. Coding regions were divided into three groups according to their Asf1-mediated gene expression (shown in shades of blue). The plot is depicted as in Figure 2C, except that the partition into groups is different. The plot shows that the link between Asf1-mediated gene expression and relative exchange holds in each independent segment of the coding region and not only in specific parts of it.

The histone chaperone Asf1 is important for disassembly and reassembly of H3/H4 histones during DNA replication, repair, and heterochromatin silencing. Asf1 is the only yeast histone chaperone that was implicated in histone H3/H4 exchange during elongation [4],[14]. Outside of replication, the contribution of Asf1 to histone H3 exchange strongly correlates with both total exchange and transcription rate [8]. The fact that Asf1 has a role in histone exchange prompted us to examine its relations with relative exchange. To that end, we used log change total exchange in wild type vs. *asf1*Δ, denoted *Asf1-mediated exchange* (data taken from [8]). The higher Asf1-mediated exchange, the higher the contribution of Asf1 to total exchange. Using this data, we have confirmed the correlation between Asf1-mediated exchange and total exchange (Figure 5A; Spearman correlation = 0.54, P-value<10^−154^, [8]).

We next sought to investigate the relations between Asf1-mediated exchange and transcription rate. In agreement with previous reports, we found that Asf1-mediated exchange indeed correlates with transcription rate (Spearman correlation = 0.25, P-value<10^−53^). However, given total exchange, the conditional correlation is insignificant (total exchange-independent Spearman P-value>0.1). This can be clearly seen in Figure 5A: in each small range of total exchange (a row in the 2D heat map), the level of Asf1-mediated exchange (color-coded) is similar along the entire row. Therefore, Asf1-mediated exchange is associated with transcription rate only indirectly, through its association with total exchange.

### Asf1-Mediated Gene Expression Is Linked to Relative Exchange

Next, we analyzed the influence of Asf1 on gene expression (i.e., transcription rate). The presence of Asf1 at promoters is important for disassembly of H3/H4 upon activation and for reassembly of H3/H4 upon loss of activation (reviewed in [2],[3],[20]). In coding regions, Asf1 travels with elongating RNAPII and influences RNAPII density [14]. Asf1 is a global transcription factor that influences transcription of hundreds of genes distributed over the entire yeast genome [15]. Asf1 has both positive and negative effect on gene expression. For example, Asf1 up-regulates transcription of SRL3 and HYR1 (confirmed by RT-PCR, [15]). On the other hand, Asf1 down-regulates PYK1, PMA1, and RPS9B (confirmed by RNAPII occupancy in promoter and coding region, [14]). This dual activity of Asf1 as transcription activator and transcription repressor is still largely unexplained.

To analyze the transcriptional influence of Asf1, we used genome-wide gene expression change in *asf1*Δ mutant vs. wild type, referred to as *Asf1-mediated gene expression* (data taken from [15], see Text S3 for details). We observe that Asf1-mediated gene expression is related to relative exchange (Spearman correlation = 0.175, P-values<10^−17^) but is not associated with total exchange (Spearman P-values>0.1; Figure 5B and 5C). The association with relative exchange appears in at least seven out of ten transcription rate bins (heat map columns in Figure 5B, Spearman correlation>0.15 in seven independent columns). Moreover, this significant association is independent of histone exchange in the corresponding promoters (see details in Text S4). We obtained the same results when the association is computed independently of GC content or transcript length (data not shown). Consistent with our observation, SRL3 and HYR1 are indeed hypo relative exchange genes, whereas PYK1, PMA1, and RPS9B are hyper relative exchange genes (data not shown). Next, we considered the possibility that the association between Asf1-mediated gene expression and relative exchange is not a consistent feature of the entire coding region. Therefore, we divided the genes into three subsets according to their Asf1-mediated gene expression. For each subset, we have plotted a profile of probe's relative exchange throughout the coding region (Figure 5D). We observe that in each segment of the coding region, the Asf1-mediated gene expression is associated with relative exchange. Therefore, Asf1-mediated gene expression corresponds to relative exchange throughout the entire coding region. While the reason for this result is yet unclear, it appears that hyper relative exchange genes are down-regulated by Asf1, whereas hypo relative exchange genes are up-regulated by Asf1.

## Discussion

In this study we investigated transcription rate–independent replication-independent histone H3 exchange in coding regions, called relative exchange (based on data from [7],[8], see Figure 1). By calculating relative exchange values, we eliminated the contribution of RNAPII transcription rate from replication-independent histone H3 exchange. Many studies investigate transcription-independent exchange, where the histone exchange is measured in the absence of transcription processes (e.g., [5]). Unlike those studies, our calculated relative exchange is not independent of transcription, but only independent of transcription rate. Therefore, relative exchange may still represent histone exchange during transcription, as long as the histone exchange is not determined solely by transcription rate.

Our analysis provides evidence that total exchange does not reflect only transcription rate. First, we show that relative exchange variability, which is independent of transcription rate, cannot be explained solely by experimental noise (Figure 2A and 2B; see corroborations in Text S2). Next, several analyses suggest that relative exchange is a feature of an entire coding region rather than a regional effect: (i) Relative exchange characterizes the entire coding region and not only a specific part of it (Figure 2C), (ii) Neighboring genes in the genome do not have a similar relative exchange (Figure 2D), (iii) relative exchange variation between neighboring genes is larger than the relative exchange variation within coding regions (Figure 2D), (iv) hyper relative exchange genes are scattered throughout the genome (Figure 3), and (v) functional enrichment tests show that hypo relative exchange genes are up-regulated by Asf1 and enriched with H3K79me3 (Figures 4 and 5, respectively). Taken together, this collection of evidence indicates that total exchange variability at the same transcription rate is a biological property of genes.

Among the numerous modified histone H3 residues, methylated H3K79 is the only one in the globular core domain, rather than in the exposed N-terminal tail. H3K79me3 occurs predominantly in the coding regions of genes and is associated with transcription activity [17]. Dot1 directly methylates H3K79 and is the main source of H3K79 methylation [21]. On the other hand, none of the identified demethylation enzyme families can remove H3K79 methylation, suggesting that H3K79 methylation might be enzymatically irreversible (e.g., [22]). This study demonstrates that relative exchange is mainly associated with H3K79me3 (Figure 4). Many possible mechanisms might explain this association. For example, H3K79me3 might be a signal for the required level of relative exchange in coding regions. Another attractive hypothesis is that histone exchange is a functional alternative to active enzymatic removal of H3K79me3. For instance, the enrichment of H3K79me3 might reflect the balance between transcription-coupled H3K79 methylation and exchange-coupled removal in each round of RNAPII transcription. If this hypothesis is correct, the slight influence of H3 exchange on the overall enrichment of H3K79me3 could be easily detected due to the simple methylation/demethylation system of H3K79 (i.e., only one methylase and probably no demethylase).

Nucleosomes are dynamically exchanged during many DNA metabolism processes, including replication, transcription initiation and elongation, DNA repair, heterochromatin silencing and basal histone exchange. Therefore, it is hard to determine the process that generates relative exchange. We assume that relative exchange is not related to replication, since the data was measured in G1-arrested cells. Several lines of evidence show that relative exchange is not established during repair or heterochromatin silencing: First, relative exchange is reproducible in different datasets (Figure 2B), and thus it is not likely to reflect a temporary cellular repair status. Second, hyper relative exchange genes are scattered in the entire genome and not localized to heterochromatin regions (Figure 2D and Figure 3). Finally, we validate that the association between Asf1 and relative exchange is independent from molecular features that are related to repair or heterochromatin silencing (Figure S5 in Text S5).

The hypothesis that relative exchange variation is generated during transcription elongation is highly attractive. Asf1 travels with RNAPII along the coding region and is the only known histone chaperone that mediates histone exchange during transcription elongation [3],[4],[14]. Since Asf1 activity is related to relative exchange (Figure 5), we hypothesize that Asf1 has a gene-specific level of activity during elongation, thereby increasing or decreasing the proportion of histone H3 exchange per RNAPII passage. This generates the observed total exchange variability across genes that have similar transcription rate. Recent reports provide evidence for specific targeting of Asf1 to promoters as part of transcription initiation [23], but to the best of our knowledge, it is still not clear whether Asf1 has a gene-specific targeting also in coding regions during elongation.

Several studies show that Asf1 has a selective positive and negative effect on gene expression, but this dual function is still largely unexplained [2],[14],[15]. In this study, we show that Asf1 has a positive transcriptional influence on hypo relative exchange genes, but negative transcriptional influence on hyper relative exchange genes (Figure 5B–5D). This provides an important insight as to the selective positive and negative transcriptional influence of Asf1. The connection might be direct, e.g., Asf1 activity might be related to RNAPII poising or a slow elongation. Alternatively, Asf1 can influence transcription rate indirectly by promoting relative exchange that removes or adds important chromatin modifications that are important for transcription. The latter alternative is supported by recent reports in several eukaryotes, demonstrating that histone exchange in promoters regulates gene expression by incorporation/removal of histone variant H3.3 (reviewed in [24],[25]). In yeast, there is no such histone H3 variant and thus detailed experiments will be necessary to reveal the precise role of gene-specific relative exchange in epigenetic transcription regulation.

## Methods

### Data Preparation

We retrieved yeast ORFs and intergenic regions from the Saccharomyces Genome Database (http://www.yeastgenome.org, July 2007). To avoid biases related to genes that are not transcribed by RNAPII and global effects on histone H3 exchange, we removed non-coding genes, 25-kbp regions near the telomeres and centromeres, and 1-kbp regions near rRNA, tRNA, ARS and mitochondrial DNA (see Text S5). In total, we applied our analysis to 3760 coding regions. Replication-independent histone H3 exchange data and RNAPII density were taken from [7],[8] (see Text S5).

### Computational Analysis

In many cases there is dependency between two molecular features x and y. To analyze x independently of confounding influences due to y, we define *y*-*independent x*-values as the distance of each x value from a running average of x values along the *y*-axis. This procedure is commonly used in noise analysis (e.g., [26]) and can be applied recursively to analyze x independently of y_1_,…,y_n_ values. For example, *relative exchange* was defined as a transcription rate–independent total exchange. Therefore, relative exchange is the vertical distance of a given total exchange point from the running average line in the total exchange–transcription rate plot (Figure 1A).

*Segmental relative exchange* was calculated as follows: we split the coding regions into six equal segments. The relative exchange of each segment was calculated using only measurements from probes located within this segment. Figure 2D presents segmental relative exchange differences between 1-st segments of different genes, and between the 1-st vs. 4-th and 1-st vs. 6-th segments of the same gene.

In Figures 2C, 4D and 5D, relative exchange values are reported per single tiling array probes from Rufiange et al. [8]. For each probe, we used total exchange and RNAPII density that were measured only on this probe (referred to as *probes' total exchange* and *probe's RNAPII density*, respectively). To calculate relative exchange, we needed the running average curve of total exchange along the RNAPII density. To avoid complications arising from different total exchange along the coding region (slightly higher near 5′ and 3′ end and lower in the middle, see [8]), we did not calculate a common running average curve for all probes. Instead, the coding regions were split into six segments of equal length, and all probes were assigned to one of six groups according to their coding region segment. All probes nearby a coding region were split into four segments (50 bp each) upstream or downstream the coding region. For each segment, we calculated a running average curve using only probes within it. The relative exchange of a probe, denoted *probe's relative exchange*, is the distance of its probe's total exchange to its segment's running average curve (on the coordinate of the probe's RNAPII density).

In order to present the relation between relative exchange and other molecular features, we used a heat map, which illustrates the functional relationship between transcription rate (*x*-axis), total exchange (*y*-axis) and an additional molecular feature (color coded; Figures 1A, 4C and 5AB). The heat map has the shape of a scatter plot, but additionally visualizes the values of the data points with respect to the third feature. In the heat map, each cell represents a 2D bin including all genes with total exchange and transcription rate in a defined range. Empty and near-empty 2D bins (below five genes) are colored white. Bins with more then five genes are color-coded according to the level of a molecular feature averaged over the genes contained within the bin. The information in each cell is therefore independent of the information in neighbor cells. In case of functional relation between relative exchange and a molecular feature, hyper exchange bins would have different color than hypo exchange bins (as illustrated in Figure 1B bottom panel).

In this study, all correlations and their P-values are based on the non-parametric Spearman correlation test [27]. The *correlation of x and z independently of y*, referred also as *y-independent Spearman correlation*, is the correlation of two variables x and z when eliminating the contribution of a third or more other variables y. This was calculated as the Spearman correlation between y-independent x and y-independent z. Therefore, we applied a non-parametric equivalent to the statistical calculation of partial correlation [27].

All reported Spearman correlations between relative (total) exchange and an additional molecular feature, were calculated independently of total (relative) exchange and transcript length. This way, the correlation with any exchange measure is calculated only after factoring out the contribution of the other exchange measure and after eliminating potential effects of transcript length. For each coding region, we used the molecular feature value averaged over the coding region. Transcript lengths were computed based on sequencing of cDNA library ([28], see Text S5 for details).

## Supporting Information

Text S1 (0.74 MB PDF)

Text S2 (0.03 MB PDF)

Text S3 (0.02 MB PDF)

Text S4 (0.04 MB PDF)

Text S5 (0.28 MB PDF)
