# Supplementary material for: Evidence for Gene-Specific Rather Than Transcription Rate–Dependent Histone H3 Exchange in Yeast Coding Regions
Source: PLoS Comput Biol. 2009 Feb 6;5(2):e1000282. doi: 10.1371/journal.pcbi.1000282 (PMC2625437; doi:10.1371/journal.pcbi.1000282)
Supplement: Text S1 — (0.74 MB PDF) [file pcbi.1000282.s001.pdf]

Text S1

We have found that the amount of relative exchange is a reproducible feature of genes (**Figure 2BC**). In the following, we confirm that this observation is not an artifact of our computational analysis.

1. In **Figure 2B**, we show that relative exchange computed based on data from Rufiange et al. (2007) strongly correlates with relative exchange computed based on data from Dion et al. (2007). For comparison, we aimed to show that experimental noise would not exhibit such high match between data from Rufiange et al. and Dion et al. To that end, we computed *replicate-independent total exchange*, i.e the distance of each total exchange to a running average on another (replicate) total exchange profile measured at the same laboratory. We hypothesize that replicate-independent total exchange (as opposed to the calculation of relative exchange where we compute transcription rate-independent total exchange) would reflect only experimental noise. **Figure S1.1** presents the relationship between replicate-independent total exchange taken from Dion et al and Rufiange et al. The clear insignificant correlation (Spearman P-value > 0.01) indeed shows that replicate-independent total exchange represents experimental noise.
2. The reproducibility of relative exchange calculated based on Rufiange et al. (2007) and Dion et al. (2007) might be an artifact or a bias in our estimation of transcription rate. To rule out this possibility, here we show that (i) the average RNAPII density is a good approximation for the actual transcription rate in steady state, and (ii) the result is not due to RNAPII poising at or nearby the TSS.
   (i) We aimed to show that RNAPII density averaged over the coding region is a good approximation for the actual transcription rate. To that end, we investigated the relationship between total exchange, RNAPII density and mRNA transcript levels (Holstege et al. 1998[1]). **Figure S1.2** shows that mRNA transcript level is indeed strongly associated with RNAPII density. Most importantly, mRNA transcript level is not biased toward hyper relative exchange (i.e., in each small range of RNAPII density, the amount of total exchange is not related to the amount of mRNA transcript level). This suggests that our results are not an artifact of using RNAPII density instead of direct measurements of transcription rate.
   (ii) RNAPII might poise at or nearby the TSS with little or no elongation. This might cause bias in our estimation of transcription rate. To rule out this possibility, here we calculated *5'-independent relative exchange* exactly as the original relative exchange were calculated, but without using information from probes located [0, +500bp] from the TSS (see **Supplement E** for details on how we estimated the TSS loci; In case of several alternative TSSs for the same gene, we used the TSS that is most proximal to the coding region. If the TSS is unknown, the translation start site was used instead). **Figure S1.3** shows that relative exchange values are similar to 5'-independent relative exchange values (Rufiange et al., Spearman correlation = 0.89). We obtained similar results when using relative exchange values and 5'-independent relative exchange values based on Dion et al. (Spearman correlation = 0.9, data not shown). **Figure S1.4** presents a clear correlation between 5'-independent relative exchange based on Dion et al. and 5'-independent relative exchange based on Rufiange et al. (Spearman correlation = 0.81). This demonstrates that the information contained in total exchange independently of transcription rate is not due to RNAPII poising at or nearby the TSS.

3. In this study, relative exchange is a transcription rate-independent total exchange that was measured 60 minutes after Flag-tagged H3 induction by galactose. The transcription rate was approximated with RNAPII density values that were measured together with the histone exchange under exactly the same treatment, 60 minutes after induction (Dion et al. 2007, Rufiange et al. 2007). One possible scenario is that hyper relative exchange genes are galactose immediate responding genes whose transcriptional response has already been finished after 60 minutes. In such case, the available RNAPII snapshot at 60 minutes does not reflect the overall mRNA production during the whole time interval. To rule out this possibility, we calculated total exchange from 60 and 90 minutes (i.e., Flag/Myc-tagged H3 difference between 60 and 90 minutes after induction; Dion et al. 2007), representing the amount of histone exchange after the early response has been finished and the RNAPII density was measured. **Figure S1.5** demonstrates the relationship between coding region RNAPII density, coding region total exchange from 0 to 60 minutes and coding region total exchange from 60 to 90 minutes. The plot clearly shows that total exchange in the first 60 minutes is in agreement with total exchange between 60 and 90 minutes, regardless the RNAPII density at 60 minutes (Spearman correlation=0.39). Therefore, in each small interval of RNAPII density after 60 minutes (a column in **Figure S1.5**), the amount of relative exchange before 60 minutes is in agreement with the amount of relative exchange after 60 minutes. We therefore conclude that relative exchange is not just an artifact of the galactose induction.
4. It is possible that the reproducibility of relative exchange (**Figure 2BC**) is due to a consistent GC content bias. However, we have found that the match between relative exchange form Rufiange et al. and Dion et al. is independent of GC content (GC content-independent Spearman correlation =0.81, P-value < 10^{-200}, **Figure S1.6**). Therefore, we conclude that the reproducibility of relative exchange is not an artifact of GC content.
5. We aimed to validate that the results are not biased by transcript length. Intergenic regions typically have high total exchange, whereas transcribed regions have progressively lower amount of total exchange such that the middle of the coding region has a lower total exchange than the 3' and 5' ends (e.g., Rufiange et al. 2007, Dion et al. 2007). Short genes therefore show higher average total exchange than long genes (**Figure S1.7A**), an important effect that might influence our results. We observe that the match between relative exchange measured in Rufiange et al. and Dion et al. is independent of transcript length (transcript length-independent Spearman correlation=0.76, P-value < 10^{-200}, **Figure S1.7B** and **Text S5**). This shows that the reproducibility of relative exchange is not an artifact of differences in transcript lengths.
6. We aimed to demonstrate the robustness of relative exchange values. Throughout the paper, relative exchange is defined as the distance from a running average curve (in this analysis, referred to as *residual* values). For comparison, *naive* relative exchange values are the coding region's total exchange divided by its transcription rate. Each of these two relative exchange approaches was applied twice on each of the two RNAPII density replicates from Rufiange et al. (denoted *residuals-1*, *residuals-2* and *naïve-1*, *naive-2*, respectively; in all cases, we used the same total exchange profile). When focusing on the 1500 genes with the highest difference between the two RNAPII density replicates, we found that the correlation between residuals-1 and residuals-2 is 0.92, whereas the correlation between naïve-1 and naïve-2 is 0.81. This result demonstrates that the residual

approach is less sensitive to noisy RNAPII density measurements than the naive approach.

7. The reproducibility of relative exchange calculated based on Rufiange et al. (2007) and Dion et al. (2007) might be a byproduct of sequence properties. To rule out this possibility, we have split each coding region into three segments of equal length. The relative exchange of a segment (denoted *segmental relative exchange*, see **Methods**) was calculated using only probes located within this segment. Probes that have an overlap with more than one segment were excluded from the analysis. The 1st-segment and mid-segment relative exchange based on Rufiange et al. and Dion et al. (respectively) could be calculated only for 952 genes. For these genes, there is a strong association between Rufiange's 1st-segment relative exchange and Dion' s mid-segment relative exchange (Spearman correlation = 0.42, P-value < 10^{-33}). Since the 1st and mid segments contain different sequences, it appears that the reproducibility between the labs is not a consequence of sequence properties.

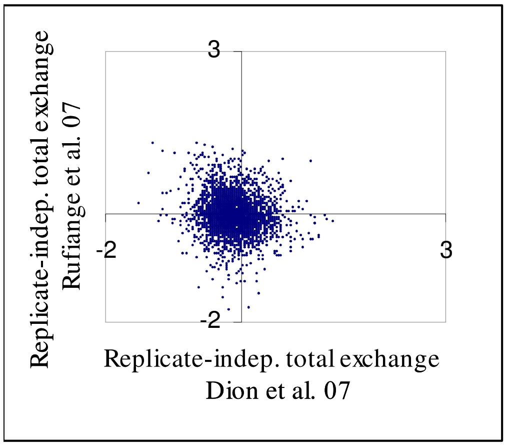

Figure S1.1: The scatter plot shows the relationship between replicate-independent total exchange taken from Dion et al (x axis) and Rufiange et al. (y axis). The clear insignificant correlation indeed shows that replicate-independent total exchange represents experimental noise.

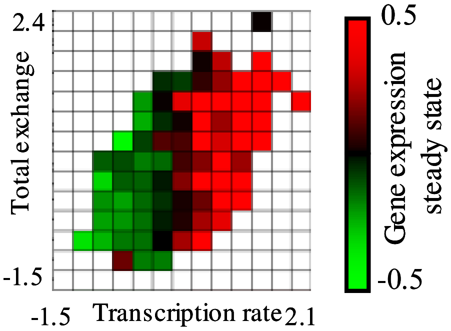

Figure S1.2: A heat map illustrating the functional relationship between transcription rate (x axis, Rufiange et al. 2007), total exchange in coding regions (y axis, Rufiange et al. 2007), and gene expression level in steady state (Holstege et al. 1998; color-coded). The heat map is depicted as in **Figure 1A,** except that the color-coding is different (see **Methods**). The green/red color-coding is according to high/low gene expression level.

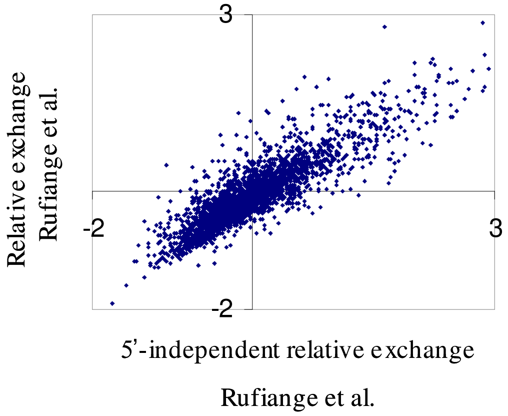

Figure S1.3: The scatter plot shows a significant correlation between relative exchange (y axis; calculated using all probes across the coding region, Rufiange et al) and 5'-independent relative exchange (x axis; calculated without information [0,+500bp] from TSSs, Rufiange et al.).

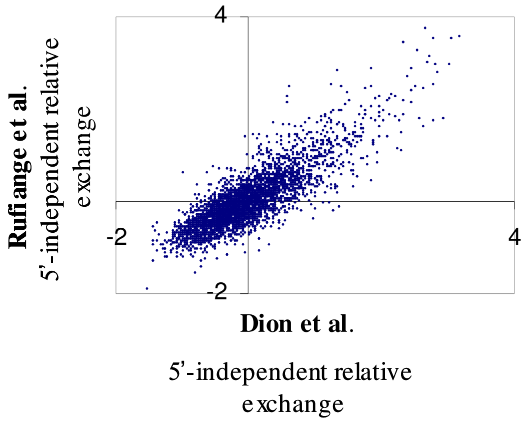

Figure S1.4: The scatter plot shows a significant correlation between 5'-independent relative exchange taken from Dion et al (x axis) and 5'-independent relative exchange from Rufiange et al. (y axis). 5'-independent relative exchange values were calculated without information from probes located [0,+500bp] from TSSs.

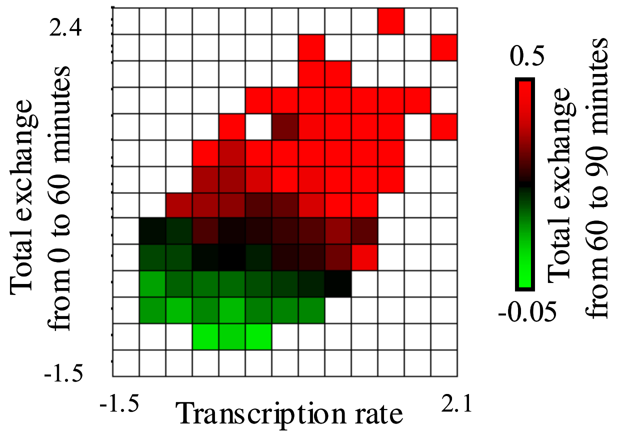

Figure S1.5: The heat map illustrates the relationship between RNAPII density at 60 minutes (x axis), total exchange from 0 to 60 minutes (y axis) and total exchange from 60 until 90 minutes (color-coded). Higher values of 60-90 total exchange values are indicated in red and lower values in green. A detailed description of the heat map presentation is in **Figure 1A** (see **Methods**). The heat map clearly shows that total exchange in the first 60 minutes is in agreement with total exchange between 60 and 90 minutes.

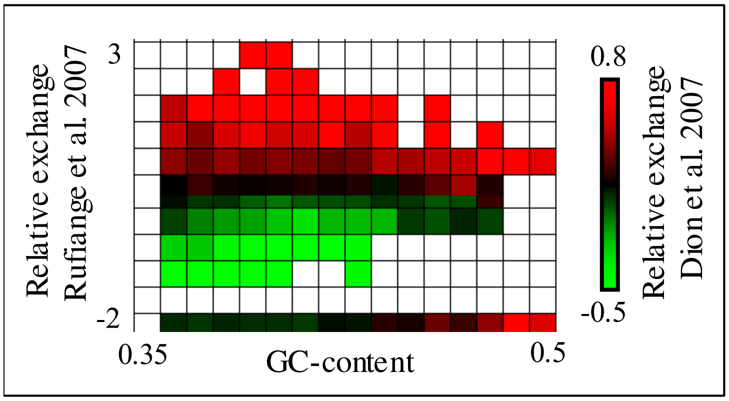

Figure S1.6: A heat map illustrating the functional relationship between average GC content in coding region (x axis), coding region relative exchange based on Rufiange et al. 2007 (y axis), and coding regions relative exchange based on Dion et al. 2007 (color-coded). High/low relative exchange values from Dion et al. are indicated in red/green. A detailed description of the heat map presentation is in **Figure 1A** (see also **Methods**). The plot shows that relative exchange based on Rufiange et al. (2007) and Dion et al. (2007) are in agreement independently of GC content.

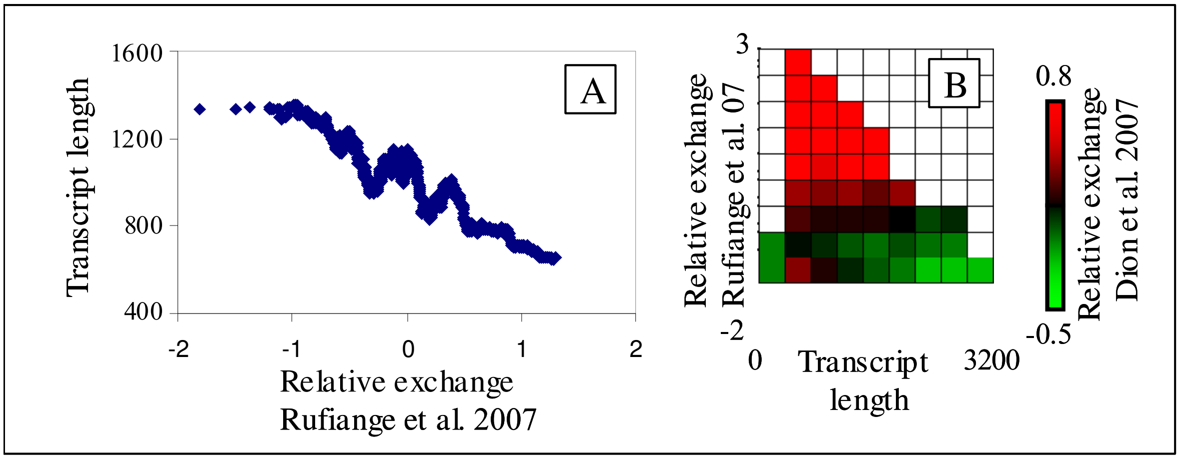

Figure S1.7: (A) Plot of the relationship between transcript length and relative exchange. The plot was generated using a moving average of a window of 100 genes. **(B)** A heat map illustrating the functional relationship between transcript length (x axis), coding region relative exchange based on Rufiange et al. 2007 (y axis), and coding regions relative exchange based on Dion et al. 2007 (color-coded). High/low relative exchange values from Dion et al. are indicated in red/green. A detailed description of the heat map presentation is in **Figure 1A** (see also **Methods**). The plot shows that relative exchange based on Rufiange et al. (2007) and Dion et al. (2007) are in agreement independently of differences in transcript lengths.

[1]Holstege FC, Jennings EG, Wyrick JJ, Lee TI, Hengartner CJ, Green MR, Golub TR, Lander ES, Young RA. (1998). Dissecting the regulatory circuitry of a eukaryotic genome. Cell **25**:717-28.
