# Supplementary material for: Evidence for Gene-Specific Rather Than Transcription Rate–Dependent Histone H3 Exchange in Yeast Coding Regions
Source: PLoS Comput Biol. 2009 Feb 6;5(2):e1000282. doi: 10.1371/journal.pcbi.1000282 (PMC2625437; doi:10.1371/journal.pcbi.1000282)
Supplement: Text S2 — (0.03 MB PDF) [file pcbi.1000282.s002.pdf]

Text S2:

The purpose of this analysis is to establish whether total exchange variation can be explained by transcription rate. To that end, we formalized a linear regression of the general form $Y = a + bX$, where Y is total exchange and X is transcription rate. Based on regression analysis (Sokal and Rohlf, 1985[1], Box 14.1) on data from Rufiange et al. (2007), 29% of the total exchange variation is explained by X, whereas 71% of the variance is the *residual* or *unexplained variation*.

Next, we extended the model as follows: $Y=a+b_1X_1 + b_2X_2$, where Y is total exchange, X_1 is transcription rate, and X_2 is relative exchange. X_1 and X_2 are independent since relative exchange is transcription rate-independent. To test this model, we used transcription rate (X_1) and total exchange (Y) values from Rufiange et al. (2007). On the other hand, relative exchange values (X_2) were taken from another laboratory (Dion et al. 2007). The covariance between X_2 and X_1 is 0.01, indicating that the two variables are independent.

If total exchange reflects only transcription rate and noise, the additional independent variable X_2 will not increase the explained variation. However, if total exchange contains information beyond transcription rate and noise, X_2 might contribute to the explained variation. Based on multiple regression analysis (Sokal and Rohlf, 1985, Box 16.1), 78% of the total exchange variation is explained by X_2 and X_1 and only 22% is still unexplained. The additional independent variable X_2 provides a significant increase in the explained variation [$P < 10^{-200}$ (F-test for significance of addition of independent variable; Sokal and Rohlf 1985, Eq. 16.14)]. Only 29% of the total exchange variation is explained by X_1, whereas 56% of the residual variation is explained by X_2, a reproducible transcription rate-independent variable called relative exchange (**Figure S2.1**).

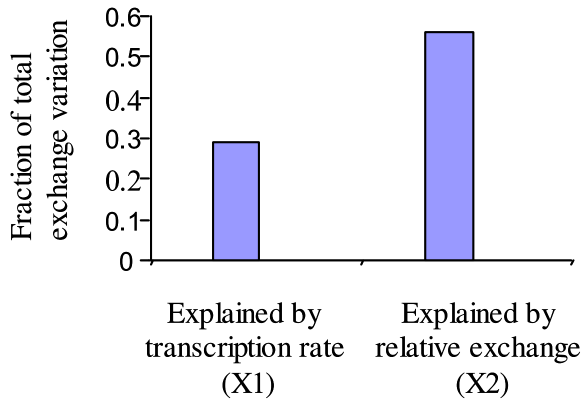

Figure S2.1: 29% of the total exchange variation is explained by transcription rate, whereas 56% of the variation is explained by the transcription rate-independent variable called relative exchange.

[1]Sokal RR and Rohlf FJ (1969). Biometry. W.H. Freeman and Company, New York.
