# Supplementary material for: Evidence for Gene-Specific Rather Than Transcription Rate–Dependent Histone H3 Exchange in Yeast Coding Regions
Source: PLoS Comput Biol. 2009 Feb 6;5(2):e1000282. doi: 10.1371/journal.pcbi.1000282 (PMC2625437; doi:10.1371/journal.pcbi.1000282)
Supplement: Text S3 — (0.02 MB PDF) [file pcbi.1000282.s003.pdf]

Text S3

Profile	Publication	Type	Channel 1	Channel 2	Additional normalization	Media	Platform
H3K14Ac	Pokholok et al. 2005	ChIP-chip	anti-H3K14Ac	anti-H3	-	YPD	60-mer tiling array
H3K9Ac	Pokholok et al. 2005	ChIP-chip	anti-H3K9Ac	anti-H3	-	YPD	60-mer tiling array
H3K36me3	Pokholok et al. 2005	ChIP-chip	anti-H3K36me3	anti-H3	-	YPD	60-mer tiling array
H3K4me1	Pokholok et al. 2005	ChIP-chip	anti-H3K4me1	anti-H3	-	YPD	60-mer tiling array
H3K4me2	Pokholok et al. 2005	ChIP-chip	anti-H3K4me2	anti-H3	-	YPD	60-mer tiling array
H3K4me3	Pokholok et al. 2005	ChIP-chip	anti-H3K4me3	anti-H3	-	YPD	60-mer tiling array
H3K79me3	Pokholok et al. 2005	ChIP-chip	anti-H3K79me3	anti-H3	-	YPD	60-mer tiling array
H3K56Ac	Rufiange et al. 2007	ChIP-chip	anti-H3K56Ac	anti-H3	-	YPD	60-mer tiling array
H3K36Ac	Morris et al. 2007	ChIP-chip	anti-H3K36Ac	anti-H3	-	YPD	ChIP DNA microarray
H3K18Ac	Kurdistani et al. 2004	ChIP-chip	anti-H3K18Ac	-	variance normalization, following Kurdistani et al.	YPD	ChIP DNA microarray
H3K23Ac	Kurdistani et al. 2004	ChIP-chip	anti-H3K23Ac	-		YPD	ChIP DNA microarray
H3K27Ac	Kurdistani et al. 2004	ChIP-chip	anti-H3K27Ac	-		YPD	ChIP DNA microarray
H3R2me2a	Kirmizis et al. 2007	ChIP-chip	anti-H3R2me2a	genomic DNA	normalized by anti-H3/genomic-DNA, following Kirmizis et al.	YPD	50-mer tiling array
Asf1-mediated expression	Zabaronick et al. 2005	gene expression	Asf1Δ strain	WT strain	-	YPD	microarray
