# Supplementary material for: Evidence for Gene-Specific Rather Than Transcription Rate–Dependent Histone H3 Exchange in Yeast Coding Regions
Source: PLoS Comput Biol. 2009 Feb 6;5(2):e1000282. doi: 10.1371/journal.pcbi.1000282 (PMC2625437; doi:10.1371/journal.pcbi.1000282)
Supplement: Text S4 — (0.04 MB PDF) [file pcbi.1000282.s004.pdf]

Text S4

Here we asked whether the activity of Asf1 in promoters plays a role in the link between Asf1-mediated gene expression and relative exchange in coding regions. For that, we analyzed together histone exchange in promoters, relative exchange in coding regions and Asf1-mediated gene expression. **Figure S4.1** demonstrates that Asf1-mediated gene expression correlates with relative exchange (in coding regions) independently of total exchange in promoters. This holds in genes with low/moderate promoter histone exchange levels (promoter total exchange < 1.5, six bottom rows in **Figure S4.1A,** see also **S4.1B**). In contrast, it seems that in promoters with high level of histone exchange, the contribution of coding region relative exchange is minor (promoter exchange > 1.5, top two rows in **Figure S4.1A**). This analysis refers only to single promoters, i.e., only intergenic regions that have exactly one downstream gene. The promoter's total exchange is averaged over 25-bp to 200-bp upstream to translation initiation (estimated translation initiation sites are taken from Miura et al. 2006[1]). We obtained similar results when we changed the intergenic regions (e.g., 25-bp until 600-bp upstream to translation initiation) or when we used only a subsection of the coding region (using only the 5' end, 3' end or the mid part of the coding regions, data not shown). We conclude that Asf1-mediated gene expression is linked to relative exchange in coding regions independently of the histone exchange in the corresponding promoters.

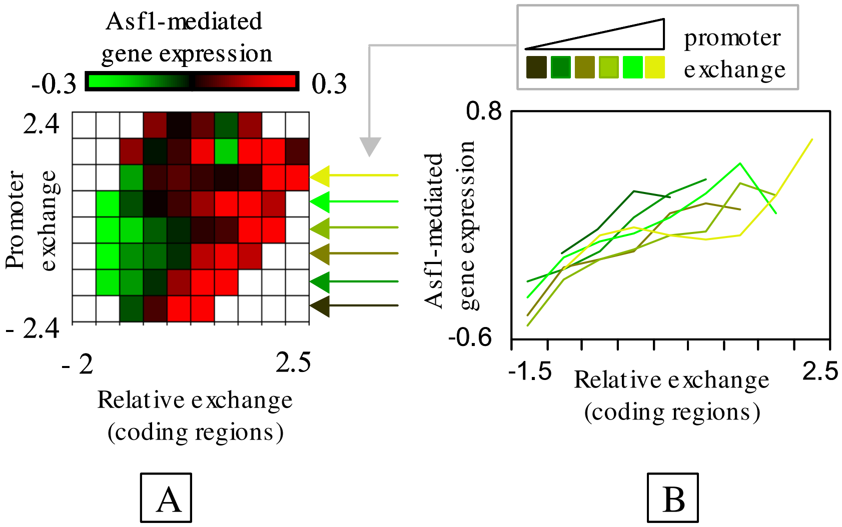

Figure S4.1: Relation between the amounts of histone H3 exchange in promoters and total exchange in coding regions. (**A**) A heat map illustrating the functional relationship between relative exchange in coding regions (x axis), the amount of histone H3 exchange in promoters (y axis) and Asf1-mediated gene expression (colo coded; Zabaronick and Tyler 2005). Down/up-regulation of Asf1 on its transcriptional targets is indicated in red/green. Low and moderate promoter exchange values are marked by arrows. **(B)** A cross-section of the heat map in A, depicting the link between Asf1 and coding region relative exchange for a fixed level of promoter histone exchange. Shown are only low and moderate promoter exchange levels, which are marked by arrows in A. The plot demonstrates that in low and moderate promoter exchange, Asf1-mediated gene expression is linked to relative exchange in coding regions independently of histone H3 exchange in promoters.

[1]Miura F, Kawaguchi N, Sese J, Toyoda A., Hattori M., Morishita S. and Ito T. (2006). A large-scale full-length cDNA analysis to explore the budding yeast transcriptome. *PNAS* **103**:17846–17851.
