# Supplementary material for: Evidence for Gene-Specific Rather Than Transcription Rate–Dependent Histone H3 Exchange in Yeast Coding Regions
Source: PLoS Comput Biol. 2009 Feb 6;5(2):e1000282. doi: 10.1371/journal.pcbi.1000282 (PMC2625437; doi:10.1371/journal.pcbi.1000282)
Supplement: Text S5 — (0.28 MB PDF) [file pcbi.1000282.s005.pdf]

Text S5

Calculating total exchange and transcription rate

Replication-independent histone H3 exchange data and RNAPII density were taken from Rufiange et al. (2007) and Dion et al. (2007). This published data was performed on a yeast strain carrying a constitutively expressed Myc-tagged histone H3, as well as an inducible Flag-tagged H3. Flag, Myc, and RNAPII were immunoprecipitated 60 minutes after Flag-H3 induction by galactose. Next, histone H3 exchange and RNAPII density were monitored on a microarray by Flag/Myc ratio and RNAPII-DNA binding, respectively. *Total exchange* is the average Flag-H3/Myc-H3 ratios in 60 minutes on all probes along an entire coding region. *Transcription rate* is the average RNAPII density in 60 minutes along an entire coding region.

Coding regions selected for this study

We retrieved 6647 yeast ORFs from the Saccharomyces Genome Database (SGD, http://www.yeastgenome.org, July 2007). We filtered the genes as follows:

1. According to the literature (e.g., Lejeune et al. 2007[1]), chromatin remodeling has an important role in centromeres and telomeres. To avoid biases related to these unique elements, we removed all genes contained within 25-kbp from each centromere and telomere.
2. Although total exchange was measured in G1-arrested cells, we find that total exchange near ARS elements is slightly elevated (**Figure S5.1, top panel**). Therefore, we removed genomic regions that are 1-kbp from ARS elements.
3. Since we analyze RNAPII, the analysis does not include rRNA and tRNA. In addition, we find that the total exchange near rRNA/tRNA is slightly elevated (**Figure S5.1, mid and bottom panel**). Therefore, we removed genomic regions that are 1-kbp from tRNAs and rRNAs elements.
4. Mitochondrial ORFs are removed.

In total, 3760 genes pass this filter and are subject to further analysis.

Estimating transcript length.

ORF length was calculated based on the SGD annotation. In addition, we adjusted the length with the distance to transcription start site (TSS). To estimate TSS position, we used sequencing of cDNA library (data available from Miura et al. 2006[2]). The library was generated by using a vector-capping method that allows the accurate mapping of TSSs. The library includes (possibly multiple) TSSs mapping for 3,599 ORFs. For each ORF, we estimated its TSS position by the average TSS mapping. Genes with no TSS mapping are excluded from all analyses related to transcript length.

.

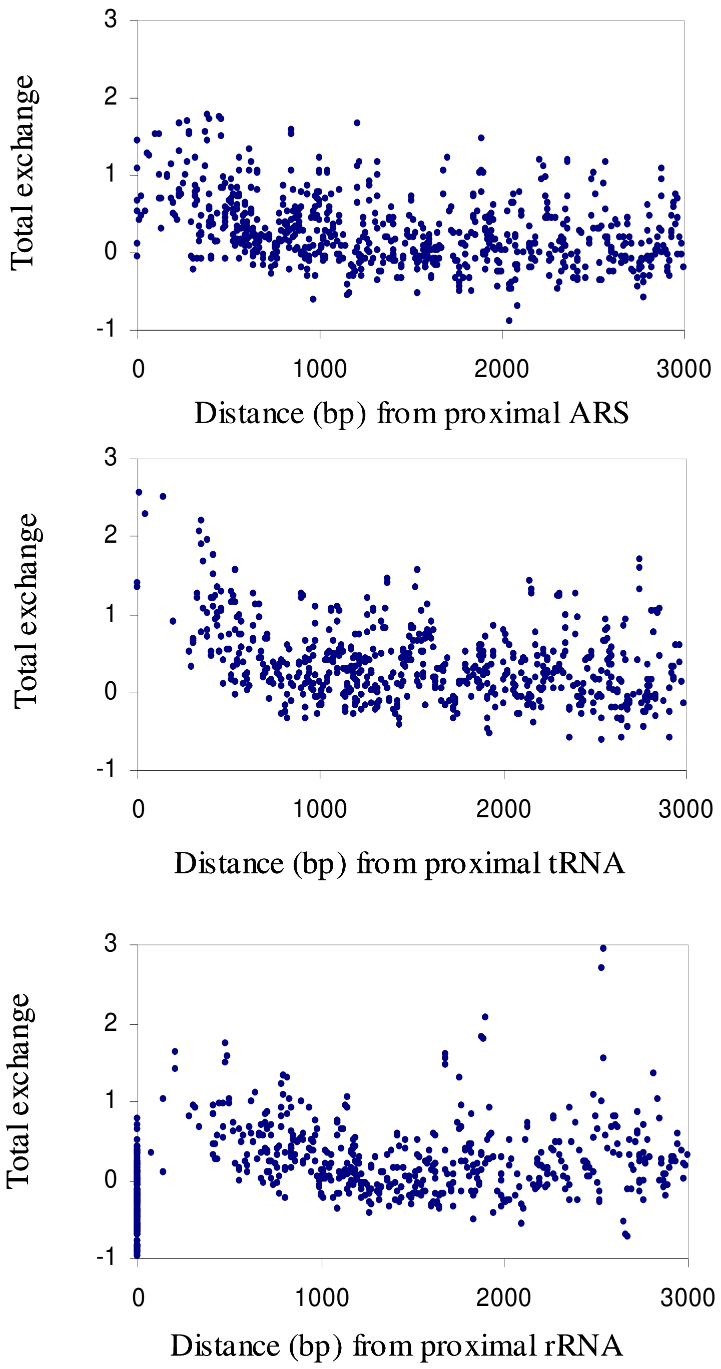

Figure S5.1: A scatter plot showing the relationship between relative exchange (y axis) and the distance (bp) from the proximal ARS, tRNA and rRNA (top, mid and bottom panels, respectively; x axis).

[1]Lejeune E. et al. (2007). The chromatin-remodeling factor FACT contributes to centromeric heterochromatin independently of RNAi. *Curr Biol* **17**:1219-24.

[2]Miura F. et al. (2006). A large-scale full-length cDNA analysis to explore the budding yeast transcriptome. *PNAS* **103**:17846–17851.
